# Denitrification Performance and Microbial Community Structure of a Combined WLA–OBCO System

**DOI:** 10.1371/journal.pone.0048339

**Published:** 2012-11-09

**Authors:** Tinglin Huang, Wei Wei, Junfeng Su, Haihan Zhang, Na Li

**Affiliations:** 1 School of Environmental and Municipal Engineering, Xi’an University of Architecture and Technology, Xi’an, Shaanxi, China; 2 Tianjin Waterworks Group Co. Ltd., Tianjin, China; Argonne National Laboratory, United States of America

## Abstract

The contamination of surface water by nitrogen due to fertilizer application and discharge of wastewater is an increasingly serious problem. A multifunctional device, which combines water-lifting and aeration (WLA) with oligotrophic biological contact oxidation (OBCO), was developed for pretreatment of raw water to reduce nitrogen. The performance of nitrogen removal and changes in microbial community structure were investigated. The results showed that the combined technique of WLA-OBCO was feasible, and that ammonium, nitrate, total nitrogen and total organic carbon were effectively removed. Meanwhile, nitrite was mostly undetectable. The PCR-DGGE and clone sequencing results revealed that α-*proteobacterium* was the largest bacterial group, and *Pseudomonas* strains Y3 and J8 were the dominant bacteria.

## Introduction

Surface water resources are often contaminated by nitrogen and organic matter due to excessive use of fertilizers and uncontrolled on-land discharge of raw and treated water, resulting in severe reductions in water quality as well as eutrophication. This limits the direct use of surface water for drinking water purposes [1]. Eutrophication in lakes and reservoirs has become an increasingly serious problem worldwide, affecting not only function and quality, but also destroying the ecological balance of bodies of water. Thus, it is necessary to remove excess nitrogen from contaminated water in order to meet criteria for usage as potable water.

Denitrification is a microbially facilitated process of nitrate reduction that may ultimately produce molecular nitrogen (N_2_) through a series of intermediate products such as NO_2_^−^, NO and N_2_O. In general, denitrification occurs where oxygen is depleted. This process is called anaerobic dentrification. However, when oxygen exists, some genera of microorganisms simultaneously use both O_2_ and NO_3_^−^ as oxidizing agents. This process is called aerobic dentrification. Much research on the dentrification of source water has been conducted, but most processes only convert nitrogen from one form to another, and fail to completely remove nitrogen from water sources [2]–[3]. Moreover, there are many problems associated with biological pretreatment of surface water sources through the use of anaerobic denitrifying bacteria.

**Figure 1:**
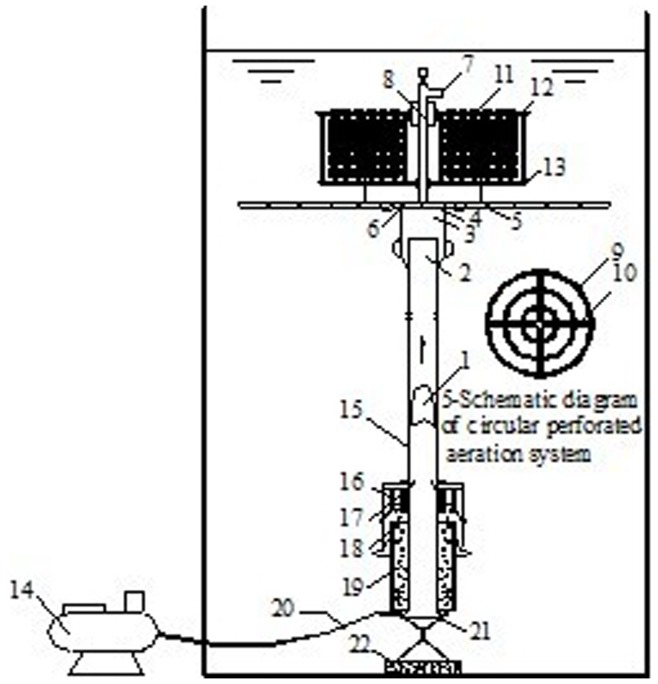
Schematic representation of the pilot experiment. 1-aeroelasticity;2-outlet;3-aeroelasticity collective chamber;4-perforated regulator plate;5-cyclic aeration system;6-pressure stabilization chamber;7-valve;8-voltage regulator pipe;9-aeration branch (DN15);10-aeration trunk (DN15);11-float;12-suspended carrier;13-filler bracket;14-air compressor;15-up-flow cylinder;16-watertight compartments;17-air chamber;18-back flow chamber;19-aeration chamber;20-inlet pipe;21-air releaser;22-anchor block.

Firstly, denitrification of surface water is mostly based on ectopic bioremediation techniques, and biological contact time is a major limiting factor for biological denitrification [4]–[10]. Secondly, most bioremediation techniques require the addition of an electron donor, such as an organic substrate, which tends to increase water treatment costs. Moreover, some studies have shown certain residual concentrations of carbonaceous compounds in the effluent, a finding that could be problematic for certain aqua-culture species [11]–[13]. Finally, water in deep layers is often found in anaerobic conditions, which tends to accelerate the release of nutrients accumulated in sediment into the water, resulting in high nutrient loading and nutrient-rich water [14]–[16]. It is difficult to fundamentally solve eutrophication with biological purification methods alone. It must combine with other technologies in order to form an efficient, economic, and stable combined technology for the purification of lakes and reservoirs.

Water-lifting and aeration (WLA) technology has been widely applied for improving the quality of source water in China [17]–[19]. This technique mixes and oxygenates water, which may facilitate the growth of aerobic dentrifiers and enhance the denitrification of water by aerobic microorganisms.

In this research, a multifunctional device, which combines water-lifting and aeration with oligotrophic biological contact oxidation (OBCO), was used for the dentrification of raw water. The main objective was to investigate the feasibility and efficiency of micropollutiant removal using the combined system with a pilot-scale in situ, which could provide a theoretical basis and technical support for field tests.

**Figure 2:**
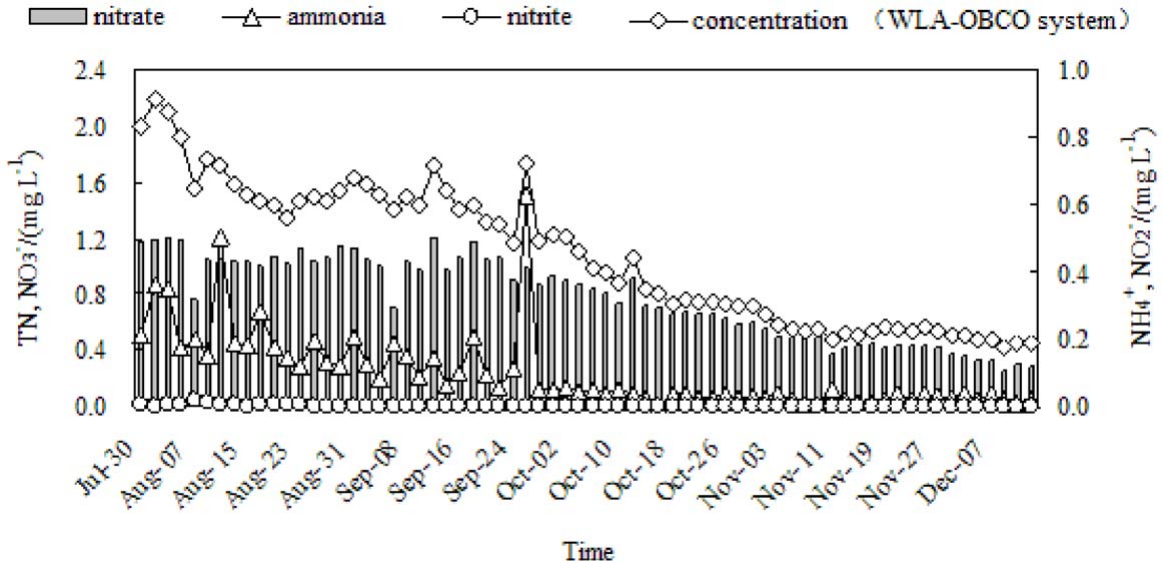
Variation of nitrogen pollutants concentrations for WLA-OBCO combined system during operation.

## Materials and Methods

### Ethics Statement

No specific permissions were required for these activities. Informed consent was obtained from all participants.

### Experimental System

#### WLA-OBCO system

A combined WLA-OBCO system is shown in Fig. 1. The WLA-OBCO system was placed in a cylindrical plexiglass container with an inner diameter of 1.8 m and a height of 4.4 m. The container was filled with 10 m^3^ of raw water to simulate natural reservoir conditions. The volume of the aeration chamber of the WLA device was 2487 cm^3^ and the up-flow cylinder had a diameter of 50 mm and a height of 3.5 m.

The air releaser, aeration chamber, backflow chamber, air chamber, up-flow cylinder, watertight compartments, gas pipeline and anchoring device were part of the WLA device, as shown in Fig. 1. The WLA device was vertically installed in the water and was fixed to the bottom of the container with an anchor block and suspended by float.

Compressed air was continuously insufflated into the air releaser as a driving force and released into the aeration chamber in the form of small bubbles. Air-mixed water was pushed into the lower water levels through the backflow chamber and local circulation was observed at the bottom layers. Gas collected in the air chamber was released into the up-flow cylinder after filling the air chamber, resulting in the appearance of a large gas bubble. The bubble pushed water up in the up-flow cylinder until it exited the device. Water in the up-flow cylinder continued to rise until the next gas bubble was formed. Water from the bottom of the container was continuously transported to the surface by the up-flow cylinder and mixed with surface water, resulting in a desirable water cycle.

The WLA device increased dissolved oxygen concentration at lower water levels through direct mixing and oxygenation. This improved water quality by creating a more suitable aerobic environment for aquatic organisms that inhibited the release of pollutants from the sediment. The biological contact oxidation system included a cyclic aeration system and suspended packing. The cyclic aeration system consisted of four aeration trunks connected with a pressure stabilization chamber and three aeration branches. Each branch contained 0.25 cm aeration holes every 4 cm. The aeration pipes were aluminum plastic pipes with a diameter of 15 mm. The biological carrier was placed in an angle bracket with a diameter of 0.4 m and a height of 0.5 m. The bracket was then placed above the perforated aeration system 0.2 m below the surface of the water. Compressed air collected in the air chamber was released, resulting in a gas bubble entering the cyclic perforated aeration pipe.

**Figure 3:**
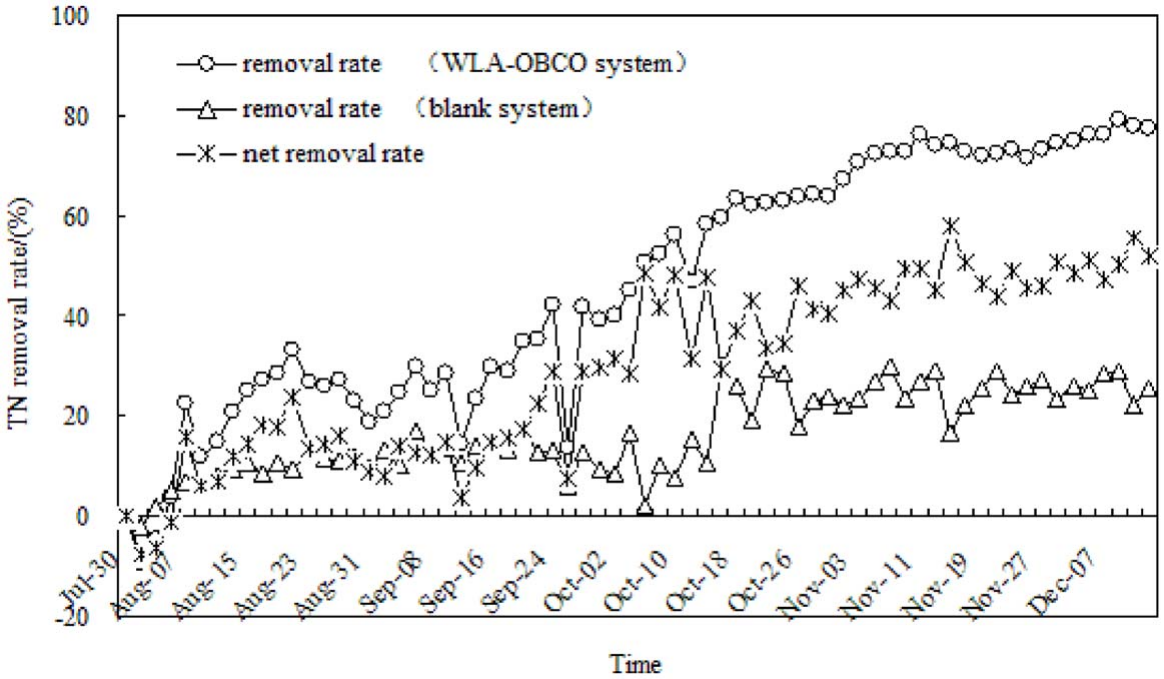
Denitrification effects of WLA-OBCO combined system during operation.

**Figure 4:**
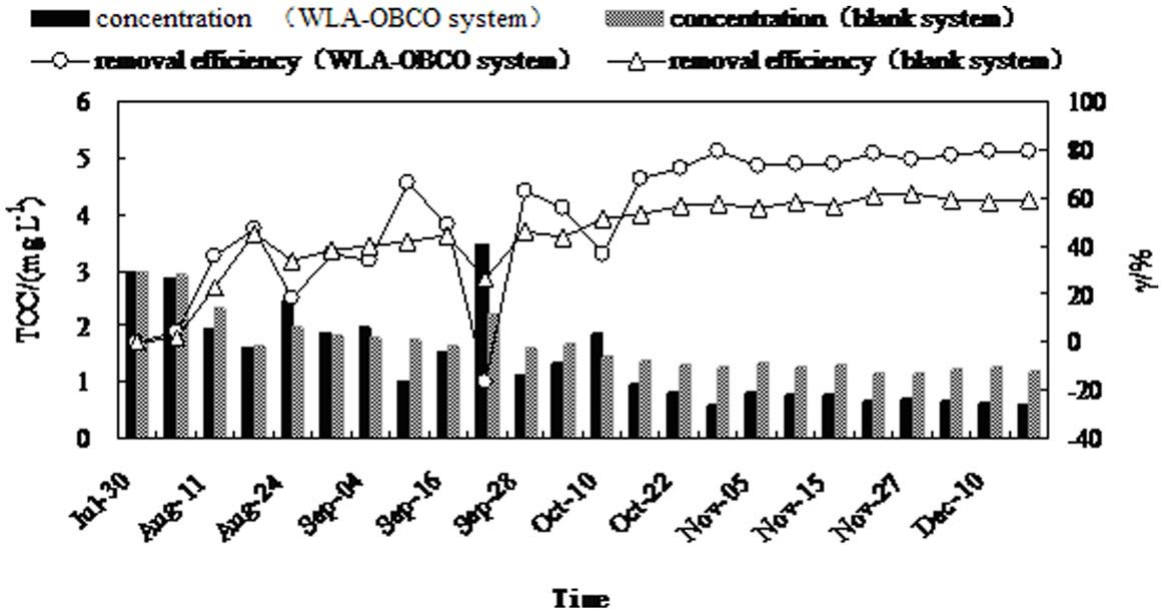
Variation of TOC concentration and removal efficiency during operation.

#### Blank system

One set of system without biological carrier and WLA device was used as the blank control group in order to compare with the purification effect of the WLA-OBCO system. The blank system was a cylindrical plexiglass container with an inner diameter of 0.45 m and a height of 4.4 m.

#### Source water

Water samples for this study were taken from a reservoir in Xi’an city, China.

#### Bacterial source and culture medium

The bacterial agents used in this study consisted of efficient aerobic denitrifiers isolated and cultured in a low-nitrogen medium in order to remove nitrogen under oligotrophic conditions.

Many studies have illustrated the difficulties of removing nitrogen from source water, owing to its low concentration as a pollutant [20]–[21]. For this purpose, a special method was used for the culturing of aerobic denitrifying bacteria in our laboratory. During two months of domestication, the target bacteria became dominant under oligotrophic and aerobic conditions. As a result, oligotrophic denitrifying bacteria were isolated and subjected to ecological combination experiments in order to obtain an optimal oligotrophic aerobic denitrifying functional bacteria group (J8, Y3, and Y7). Through analysis of their physiological and biochemical properties, and through sequence analysis of 16S rDNA, strain J8, Y3, and Y7 were identified as *Pseudomonas* sp. The pure strains of J8, Y3, and Y7 were inoculated individually into a denitrification medium solution and cultivated under conditions of rotary shaking at 120 rpm, temperature at 30°C, and an incubation period of 24 h. The above bacterial suspensions with 2% of total inoculation volume were placed into sterilized raw water for the adaptive culture. The pre-culture that was obtained was then used for biofilm cultivation.

Denitrification culture medium (g L^−1^): CH_3_COONa 0.1, NaNO_3_ 0.02, K_2_HPO_4_ 0.02, MgCl_2_ 0.01 and CaCl_2_ 0.01, pH 7.0–7.5.

### Biological Packing Material

The biological packing material was mainly made of polypropylene and polyethylene. This material had the advantages of having both a high specific surface area and hydrophilic properties, which favored the development and establishment of microorganisms. The suspended carrier had a specific gravity of about 1 after inoculation and rotated freely under the aeration and could make good use of the oxygen by colliding and cutting the air bubbles. Indicators of physical and chemical properties were as follows: diameter 10 mm; height 10 mm; bulk density 150 kg m^3^; specific surface area more than 500 m^2^ m^−3^; porosity about 75%; application temperature −35°C to 65°C; and accumulation number 760000 m^−3^.

**Figure 5:**
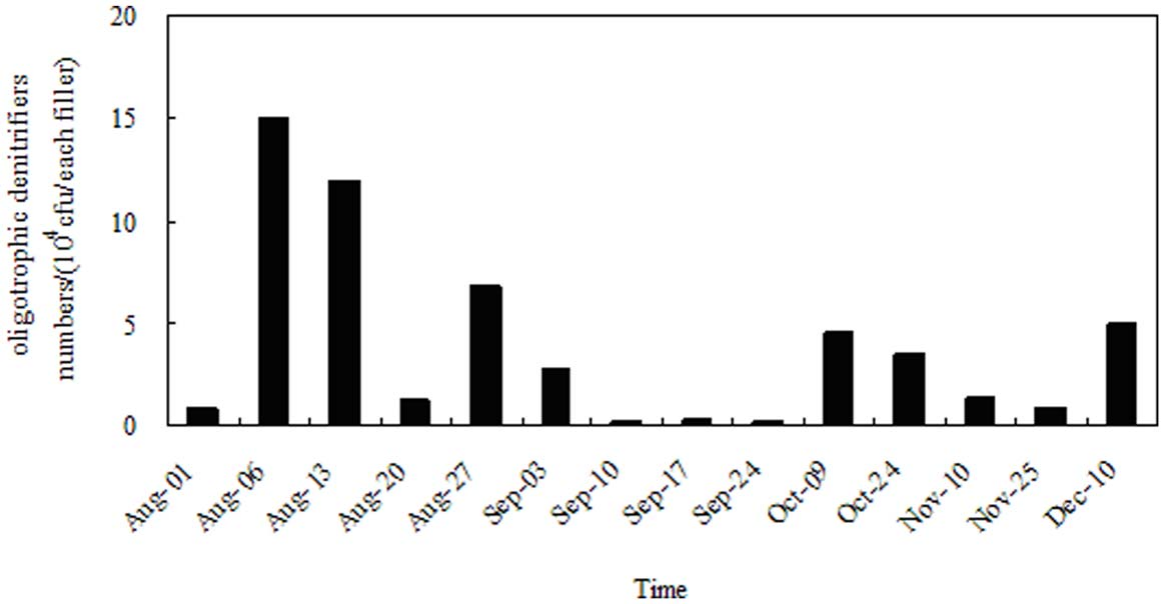
Changes of oligotrophic denitrifiers number on the fillers during operation.

**Figure 6:**
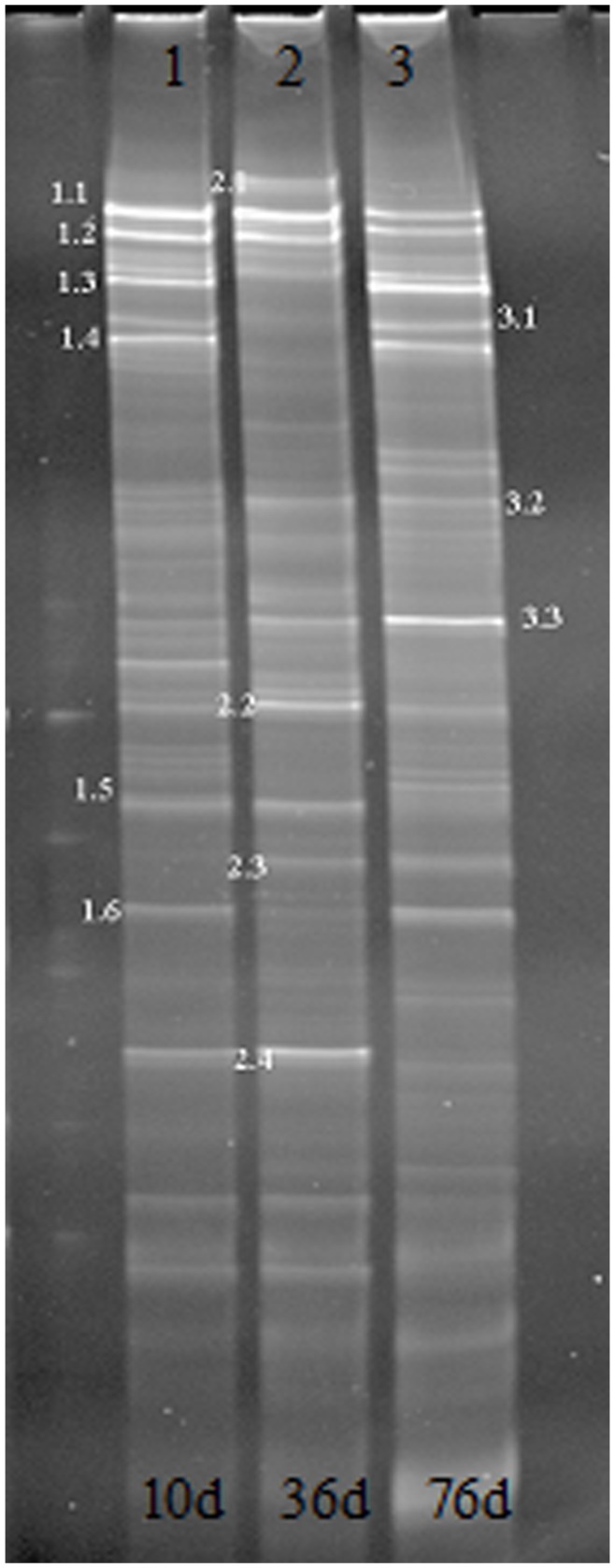
DGGE profiles of the 250-bp PCR fragment of 16S rDNA genes (V3 region) amplified from different biofilm samples.

### Analytical Equipment and Methods

NH_4_-N, NO_2_-N, NO_3_-N, and TN concentrations were determined according to the standard method using an ultraviolet spectrophotometer (HACH, Model DR5000, USA) (CEPA, 2005). Temperature and pH values were measured with a glass electrode pH meter (EUTECH, Model pH510, USA). DO concentration was estimated by a DO meter (HACH, Model HQ-30d, USA). TOC concentration was determined by IL500 type TOC analyzer (HACH Company, USA).

Biofilm samples were observed under BX51 fluorescence microscope (OLYMPUS Company, Japan) and TS5136XM scanning electronic microscope (VEGA Company, German). The dilution plate method was employed for counting oligotrophic denitrifying bacteria cultured in denitrification solid medium for 168 h at 28°C.

### Determination of Microbial Community Structure

#### DNA extraction and purification

Bacterial DNA was extracted following the method of Tsai and Olson [22], with slight modification. 30 ml of biofilm suspension was filtered with a polycarbonate filter (Millipore, pore size 0.2 µm) to collect bacteria cells. The filter was added to 5 ml of TE buffer (0.1 M Tris·Cl, 0.1 M EDTA-Na_2_, 0.2 M NaCl, 2% CTAB, pH 8.0) and incubated at 37°C for 45 min with agitation (100 rpm). 0.75 ml of 20% SDS (w/v) was added and followed by a water bath at 65°C for 1 h. These samples were centrifuged for 10 min at 12000×*g*. The supernatant was transferred to a new tube and further extracted twice with an equal volume of phenol-CIAA (phenol:chloroform:isoamylalcohol, 25∶24∶1). Finally, nucleic acids in the extracted supernatant were precipitated with sodium acetate (final concentration 0.3 M, pH 5.2), and 2.0 volumes of 100% ethanol for 1 h at room temperature. The pellet of crude nucleic acids was obtained by 20 min of centrifugation at 12000×*g*. The pellet was washed with 70% ethanol, dried for 10 min under a vacuum and dissolved in 50 µl TE buffer.

#### Primers and PCR amplification

The V3 variable region of bacterial 16S rDNA was amplified using two primers described by Muyzer et al [23]: F338-GC (5'-CGCCCGCCGCGCGCG GCGGGCGGGGCGGGGGCACGGGGGGCCTACGGGAGGCAGCAG-3') and R518 (5'-ATTACCGCGGCTGCTGG-3'). A GC clamp of 40 nucleotides was added to the forward primer 5' of F338 in order to ensure that DNA fragments would remain partially double stranded [24]. PCR reaction system (50 µl) included 0.5 µl 100 ng of template DNA, 0.25 µl of Taq polymerase (5 U), 1 µl of primers F338-GC, 1 µl of R518 (10 µM), 5 µl of tenfold PCR buffer (containing 2.0 mM MgCl_2_), 1 µl of dNTP (10 mM), and 41.25 µl of UV-sterile water. The following PCR program was used: an initial denaturation at 94°C for 4 min, followed by 30 cycles of 94°C for 30 s, 56°C for 1 min, 72°C for 30 s, and a final extension at 72°C for 7 min. The final extension for 7 min was performed to eliminate the occurrence of artificial double bands in subsequent DGGE analysis [25].

**Table 1:** Closest relative species or isolated oligotrophic denitrifying bacteria of the selected clones (Determined by an NCBI BLAST search).

Band	Sequence length/bp	The bacterial species searched in GenBank or isolated oligotrophic denitrifying bacteria having the closest relationship with the dominant bands(Base sequence similarity)
1.1	193	*Eubacterium* WD229; AJ292593(92%)
1.2	195	J8(100%)
1.3	194	*Beta proteobacterium* HTCC304; AY429720(96%)
1.4	194	*Beta proteobacterium*; Z32M51B; FJ484386(100%)
1.5	169	*Magnetospirillum* sp.; MSM-4; Y17390(94%)
1.6	169	Uncultured bacterium; JG35-K2-AG47; AM116752(92%)
2.2	169	*Mesorhizobium* sp. Acj 104; AB480752(97%)
2.3	174	Uncultured bacterium; FC04A09; FM873233(99%)
2.4	174	Uncultured bacterium; nbu202c02c1; GQ020041(100%)
3.1	193	Y3(100%)
3.2	194	*Pseudomonas geniculata* (T); ATCC 19374T; AB021404(100%)
3.3	194	*Comamonas testosteroni*; Q10; AF519533

#### Denaturing gradient gel electrophoresis (DGGE)

The expected size of the amplified fragment was 250 bp. DGGE analysis was performed in a DGGE apparatus (Bio-Rad, Richmond, CA, USA). Approximately 3 µl of PCR products were loaded onto an 8.0% (*w*/*v*) polyacrylamide gel cast in 1×TAE buffer. The polyacrylamide gels (acrylamide:bisacrylamide, 37.5∶1) were made with denaturing gradients ranging from 30 to 60%. 100% denaturant contained 7 M urea and 40% formamide. Electrophoresis was carried out at 60°C with a voltage of 150 V in 1×TAE buffer for 4 h. Bands were visualized using a UV transilluminator after staining the gel with ethidium bromide (EB) and photographed.

#### Cloning and sequencing

The dominant bands in the DGGE gel were excised. Each excised piece was washed twice with 1 ml of sterilized distilled water. A small chip (less than 1 mm^3^) of each piece was used as a direct template for PCR to recover the DNA fragment.

The selective bands on the EB-stained DGGE gel were assigned to different species after their isolation, reamplification by PCR, and sequencing. The selective bands were incised and then placed in 1.5 ml tube to reclaim the DNA. DNA was reclaimed using DNA reclaim kit (Shanghai Sangon: SK1135) according to the manufacturer’s instructions. The reclaimed DNA was used as a template to reamplify the bands with the same pair of primers (not containing the GC clamp) and the same PCR conditions as described earlier. Amplicons were then purified by SK1191 UNIQ-10 DNA Gel Extraction Kit (Shanghai Sangon) according to the manufacturer’s protocol and sequenced with one of the amplification primers. These sequences were finally compared with similar sequences in the Genbank DNA database using BLAST analysis (Basic logical alignment search tool, BLAST at NCBI) [26].

#### Operating conditions

Samples were taken 1.7 m from the bottom of the plexiglass container due to the mixing effect of the WLA system. Source water was purified by the WLA-OBCO system under conditions of dissolved oxygen and temperature 10°C∼23°C. All pollutant removal efficiencies were calculated at the cumulative removal rate because there were no influent and effluent pollutants in the container.

Biofilm cultivation could be accelerated artificially by adding bacteria liquid according to the method in section 2.3. The pre-culture obtained above was added to a sterile liquid medium and incubated at 30°C for 2–3 d. Fillers were then placed into the cultures. Microorganisms were cultured under conditions of aeration, and attached to the biological packing materail for the growth and reproduction. The biofilm was gradually forming on the biological packing materail after a few days. The fillers with biofilm were then set into the bracket fixed in pilot experimental system.

## Results and Discussion

### Denitrification Effects of WLA-OBCO Combined System

The WLA-OBCO combined system showed a desirable denitrification effect for source water under conditions of COD_Mn_/TN 1.56, temperature 10°C∼23°C, and dissolved oxygen 5.0∼7.0 mg L^−1^. Since aerobic denitrifiers were used in the system, the denitrification process could not be inhibited by oxygen. NO^3−^ and O_2_ could be used as the final electron receptors and reduced at the same time. Therefore, NO^3−^ still could be reduced to nitrogen gas or other gaseous oxides of nitrogen by the microorganisms in the combined system.

The experimental results for ammonium, nitrate, nitrite and total nitrogen concentration variations are illustrated in Fig. 2. Nitrogen pollutants were adsorbed quickly by the biofilm at the beginning of the experiment, resulting in a slight decrease of ammonium, nitrate and total nitrogen concentrations on the 8th day. However, for approximately 2 months after beginning operation of the WLA-OBCO system, nitrate and total nitrogen removal rates increased slowly (in the range of 5%∼20% and 10%∼35%, respectively), which suggested that the bacterial population adapted to the low nutrition environment over a long period of time. The microorganisms acclimatized themselves to the diminished nutriment level by reducing their metabolic levels, extending their specific areas, and utilizing a larger number of substrates. During this period, the morphology and physiology of oligotrophic bacteria experienced a significant change, resulting in a steady starvation-survival state with cell size remaining almost constant. Additionally, the metabolic efficiency of the microorganisms improved due to energy deficiency [27].

Rapid removal of nitrogen pollutants followed these adaptations. The removal rates of ammonium, nitrate and total nitrogen in a steady running period ranged from 82%∼100%, 62%∼79%, and 71%∼80%, respectively. Since total nitrogen concentration was only 0.418 mg L^−1^ during the late experiment period, less than the limits of 1.0 mg/L for class III surface water quality according to GB3838-2002. These results indicate the necessity of establishing a longer biological contact time for enhancing denitrification efficiency, one of the most attractive advantages of the WLA-OBCO combined system. The nitrite concentration changed little during the entire operation period and was not detected after November 3, 2009. Thus, the unique design of the WLA-OBCO system ensured dependable water quality with regard to ammonium, nitrite, nitrate and total nitrogen.

The removal rate and net removal rate of total nitrogen for the WLA-OBCO combined system can be shown in Fig. 3. The total nitrogen removal rate of WLA-OBCO system was almost always higher than that of the blank system during the entire operation. The total nitrogen removal rate of blank system was only 29% when the total nitrogen removal rate of WLA-OBCO system reached the maximum of 79%, the net total nitrogen removal rate of 50% was obtained for the WLA-OBCO system. These results show that the desirable denitrification performance can be obtained absolutely under the combined effects of the biodegradation of OBCO system and the aeration of WLA.

### The Effect of Water Treatment on TOC

Total organic carbon (TOC) was often used to evaluate the degree of organic pollution in the water. As shown in Fig. 4, a desirable removal efficiency of TOC was achieved during the entire operation. The consumption of TOC mainly resulted from the metabolic activities of microorganisms such as oxidation, reduction and synthesis, and the biological flocculation and adsorption process. Biodegradable organic matter served as electron donors and carbon sources for heterotrophic bacteria and was beneficial for the propagation of oligotrophic microorganisms. The biofilm applied in micro-polluted raw water was mainly composed of oligotrophic bacteria, which helped consume and remove organic matter from the surrounding environment. Fig. 4 and Fig. 2 show that the trend for organic matter and total nitrogen concentrations were similar, since oligotrophic denitrifiers could use organic matter as electron donors and use carbon sources in denitrification and cell synthesis. Therefore, the denitrification effect had a direct relationship with organic matter. The degradation of organic matter was not obvious and TOC removal efficiency was in the range of 18% to 37% between July 30 and August 29, 2009. The metabolic efficiency of oligotrophic microorganisms improved following adaptation of the biofilm system to the source water. Consequently, efficiency of organic matter utilization also increased. The organic matter removal rate was higher than 70% during the stable operation phase, reaching a maximum removal rate of 80% for TOC, and more than the TOC maximum removal rate of 57% for the blank system. TOC concentration fell from an initial 2.979 mg L^−1^ down to 0.609 mg L^−1^. These results also show a sharp increase in TOC concentration to 3.46 mg L^−1^on September 22, 2009. This increase seemed to have been caused by a lack of organic matter, which led microbial cells on the fillers to develop into an endogenous metabolic phase, which consequently resulted in death or hydrolysis, and an increase of organic matter. However, this part of the organic matter was mostly biodegradable and was beneficial for biological denitrification.

### Changes of Oligotrophic Denitrifying Bacteria

Fig. 5 summarizes the changes in the number of oligotrophic denitrifying bacteria on the biofilm during the entire experimental period. When organic nutrients in the raw water were relatively abundant, oligotrophic denitrifying bacteria used organic carbon as energy and as electron donors for the purpose of achieving microbial growth, removal of organic and nitrogen pollutants in the process of metabolism, and synthesis of cell and denitrification. As indicated in Fig. 5, the number of oligotrophic denitrifying bacteria could increase on the order of 10^3^ to 10^5^ cells per filler owing to relatively abundant sources of carbon during the early part of the operation. As the experiment ran on and as nutrients in raw water were consumed, the microorganisms would utilize their own stored nutrients through endogenous respiration. However, as stored nutrients were exhausted, the cells began to die or break down, causing the number of denitrifying bacteria to decline to (2.0∼3.6)×10^3^ cells per filler during the second month. Fig. 4 shows the TOC value suddenly increasing in late September due to the death of some bacteria. The remaining bacteria would continue to use part of this organic matter as a substrate for growth and reproduction, allowing the number of denitrifying bacteria to increase to 4.5×10^4^ cells per filler in early October. In the middle and late period, the microorganisms gradually adapted to the oligotrophic environment, with the structure of the biological community on the biofilm trending stable. The number of denitrifying bacteria changed little, with bacteria growth and death at a state of dynamic equilibrium.

### Diversity of Microbial Community Structure Analysis

Samples taken from the biofilm in different periods were prepared for DGGE analysis. DGGE separation of PCR products were isolated for different electrophoretic bands on different locations, and were used to identify the diversity of the microbial community structure and biological diversity for different samples.

The diversity of bacterial community in each sample was studied by PCR-DGGE analysis of amplified V3 region of 16S rDNA genes. The DGGE banding patterns showed that the number and intensity of migrating bands of the DNA profiles of all samples were changeable during the operation (Fig. 6). Each sample showed a specific profile. Some bands disappeared, and new bands appeared during the operation, indicating not only the demise of the original species, but also the growth of new species. Twelve bright and representative main bands were selected for tapping, DNA elution, recycling, re-amplification and electrophoresis after isolation. Each re-amplified band was purified, underwent ligation, transformation, cloning, and sequencing. Homology analysis of sequences was made by searching in GenBank with NCBI-BLAST in order to find the bacteria having the closest relationship with each sequence, as shown in Table 1.

The twelve dominant bands analyzed could be divided into α-*proteobacterium*, β-*proteobacterium*, γ-*proteobacterium* and *Actinobacteria*s. However, α-*proteobacterium* and β-*proteobacterium* were the two main bacterial groups, constituting 33% and 25% of all bands, respectively. Bacteria belonging to the *proteobacteria* accounted for 75% of all bands. Some studies have shown that the α-*proteobacterium* outline contains many bacteria genera that have a high adaptability to nutrient-poor environments, thus it was expected that α-*proteobacterium* would appear in the biological pretreatment system for drinking water. Chen et al. studied the microbial community structure for drinking water treatment using a reverse osmosis membrane reactor using 16S rDNA clone library and FISH methods, and the results of these two methods consisted with the findings that the bacteria belonging to α-*proteobacterium* could account for 50% of all bacteria [28]. Another bacterial group appearing in the tapping bands was β-*proteobacterium,* one of the major groups of the bacteria domain. Studies have shown β-*proteobacterium* being the most dominant group in wastewater treatment systems. However, β-*proteobacterium* was not the largest group in our experimental results, probably due to variations in the bacterial community structure owing to differences in the types and concentrations of pollutants in water supply vs. wastewater treatment. Sequencing analysis indicated that two main types of nitrifying bacteria *Nitrosomonas* and *Nitrospira* did not appear in the tapping band due to the low concentration of ammonium in the raw water.

In addition, the sequence of band 1.2 was 100% identical with that of strain J8, and the sequence of band 3.1 showed 100% similarity to the gene of strain Y3 by sequencing and NCBI analysis. These results imply that there were other dominant bacteria–other types of aerobic denitrifiers or other bacteria more well-adapted to the environment–in the biofilm system beside the oligotrophic denitrifying bacteria. These may form a stable system by synergic competition effects to remove the nitrogen and organic pollutants.

### Conclusions

(1) Pilot research on the WLA-OBCO combined process showed potential for the system as an alternative for drinking water purification, as it reduced the risk of nitrogen contamination and did not require high operational costs. The removal rates of ammonium, nitrate, total nitrogen and TOC in steady running period ranged from 82%∼100%, 62%∼79%, 71%∼80% and 73%∼80%, respectively, under the conditions of oxygen and temperature 10°C ∼23°C for source water. Nitrite could not be detected. The nitrogen removal effects could meet the requirements of class III of surface water quality according to GB3838-2002.

(2) The number of oligotrophic denitrifying bacteria on the biofilm changed regularly during running period. Oligotrophic denitrifying bacteria number could increase to the order of 10^4^ to 10^5^ cells per filler when carbon source was relative abundant in raw water. Denitrifying bacteria number changed little and bacteria growth and death was basically in a state of dynamic equilibrium in steady operation.

(3) The PCR-DGGE profiles showed that the number and intensity of migrating bands of the DNA profiles of all samples were changeable. Sequencing results revealed that α-*proteobacterium* was the largest bacterial group, and strains J8 and Y3 became the dominant bacteria and played their role to achieve the purpose of water purification with other bacteria.
